# Using the Stop Transmission of Polio (STOP) Program to Develop a South Sudan Expanded Program on Immunization Workforce

**DOI:** 10.1093/infdis/jiw563

**Published:** 2017-06-30

**Authors:** Dieula D. Tchoualeu, Margaret A. Hercules, William B. Mbabazi, Anthony L. Kirbak, Abdulmumini Usman, Ketema Bizuneh, Hardeep S. Sandhu

**Affiliations:** 1 Global Immunization Division, Center for Global Health, US Centers for Disease Control and Prevention, Atlanta, GA;; 2 African Field Epidemiology Network, Kampala, Uganda; and; 3 South Sudan Ministry of Health,; 4 World Health Organization South Sudan, and; 5 United Nations Children’s Fund South Sudan, Juba

**Keywords:** Global Polio Eradication Initiative (GPEI), transition, legacy, workforce development, human resources, Republic of South Sudan, Expanded Program on Immunization (EPI), capacity building.

## Abstract

In 2009, the international Stop Transmission of Polio (STOP) program began supporting the Global Polio Eradication Initiative in the Republic of South Sudan to address shortages of human resources and strengthen acute flaccid paralysis surveillance. Workforce capacity support is provided to the South Sudan Expanded Program on Immunization by STOP volunteers, implementing partners, and non-governmental organizations. In 2013, the Polio Technical Advisory Group recommended that South Sudan transition key technical support from external partners to national staff as part of the Polio Eradication and Endgame Strategic Plan, 2013–2018. To assist in this transition, the South Sudan Expanded Program on Immunization human resources development project was launched in 2015. This 3-year project aims to build national workforce capacity as a legacy of the STOP program by training 56 South Sudanese at national and state levels with the intent that participants would become Ministry of Health staff on their successful completion of the project.

Decades of civil war in South Sudan have resulted in the collapse of its infrastructure and public health system. The impact of this war on the immunization program was evident when the diphtheria-tetanus-pertussis (DTP) coverage was documented at 10% in 2004 [1]. The signing of the comprehensive peace agreement between the government of Sudan and the Sudan People’s Liberation Movement on 9 January 2005 ceased the civil war that plagued the country for decades. On 9 July 2011, the Republic of South Sudan became the youngest country to have gained full independence.

After the signing of the comprehensive peace agreement, the government of South Sudan began to rebuild its healthcare system. In 2006, it released its “Health Policy for the Government of Southern Sudan,” which provided early guiding principles for the restructure of South Sudan’s public health system [1]. The South Sudan Ministry of Health (MOH) followed suit with its “Basic Package of Health and Nutrition Services for South Sudan,” which includes immunization services among the main health priorities in the government’s health agenda [2]. The 2007–2011 comprehensive multi-year plan (cMYP) and immunization policy were developed by the MOH and its partners to provide strategic guidance on planning and implementing immunization activities in South Sudan [1, 3].

Through multilateral efforts, the DTP3 coverage rate for children <1 year old, as shown by administrative data, improved from 10% in 2004 to 22% in 2008 and then to 71% in 2010, with a decline in DTP1-DTP3 dropout rate from 41% to 27% over the same period [1, 4]. South Sudan reported its last indigenous case of wild poliovirus (WPV) in 2002. However, the country has since experienced several WPV outbreaks due to importation—8 cases in 2004 (6 WPV type 1 and 2 WPV type 3); 4 WPV type 3 cases in 2005; 24 WPV type 1 cases in 2008; 40 WPV type 1 cases in 2009; 2 circulating vaccine-derived poliovirus type 2 cases in 2014; and 1 vaccine-derived poliovirus type 2 case in 2015 [5].

The importation of WPV was most likely due to massive displacement of refugees during the civil war and low routine immunization (RI) coverage. Implementation of supplemental immunization activities (SIAs), including national immunization days and mop-up campaigns, contributed to South Sudan’s being free of WPV transmission since June 2009. This achievement and a need to update the cMYP for 2012–2016 led to an external comprehensive review of the Expanded Program on Immunization (EPI) in 2011 to document key milestones achieved and lessons learned over the years and to identify weaknesses and threats to the EPI.

Despite major challenges in the country, results of the 2011 EPI review revealed several accomplishments of the program (ie, improvement in immunization coverage, interruption of WPV outbreaks, and more). However, major gaps and challenges were also noted. One key challenge documented was the paucity of qualified staff working on immunization-related activities within all levels of the healthcare system. More than 80% of required EPI positions at national level and 50% of required EPI positions at the state level were vacant [4]. Very few staff had received training on immunization practice within 2 years before the 2011 comprehensive external EPI review. Most health facilities were not operational at the payam [district] level, and many were supported by non-governmental organizations rather than the MOH. Furthermore, the World Health Organization (WHO) and the United Nations Children’s Fund (UNICEF) provided most of the workforce capacity to the South Sudan EPI, including State EPI focal points (WHO), temporary international focal points (WHO), cold-chain officers (UNICEF), communication for development officers (UNICEF), and the Stop Transmission of Polio (STOP) volunteers (WHO).

## ROLE OF THE STOP PROGRAM IN SOUTH SUDAN

The STOP program is one of the pillars of the Global Polio Eradication Initiative (GPEI). It was conceptualized and implemented in 1999 by the US Centers for Disease Control and Prevention (CDC) in collaboration with WHO to address shortages of human resources and to strengthen technical assistance for polio eradication and other immunization activities in polio-endemic countries [6]. The program was later expanded to include measles mortality reduction, RI, communications, and data management capacity strengthening.

STOP is a volunteering program that recruits master’s and/or doctoral level public health professionals with ≥5 years of work experience in capacity building; vaccine-preventable disease (VPD) surveillance for acute flaccid paralysis (AFP), measles, and rubella; health communication and/or social mobilization; and health information systems, specifically with immunization and VPD surveillance data. Under the leadership of WHO, STOP began supporting the GPEI in South Sudan in 2009 by deploying 20 international volunteers to conduct field assignment for a period of 4–6 months. Continuous deployment of STOP volunteers in the same region or area of the country has been the practical norm. The STOP volunteers are allowed to do a maximum of 4 assignments in the program.

From 2009 until present, a total of 57 STOP volunteers were deployed in South Sudan to focus on core areas of the EPI. Of this number, 41 field epidemiologists were deployed on 203 assignments, 5 data managers on 28 assignments, and 11 communication specialists on 21 assignments. These STOP volunteers were placed to fill gaps and provide overall support to the local EPI. Over the years, they have established and supported community-based surveillance, implemented polio national immunization days, supported RI and SIAs, improved data management and quality, and provided other immunization-related activities as needed.

**Figure 1.**
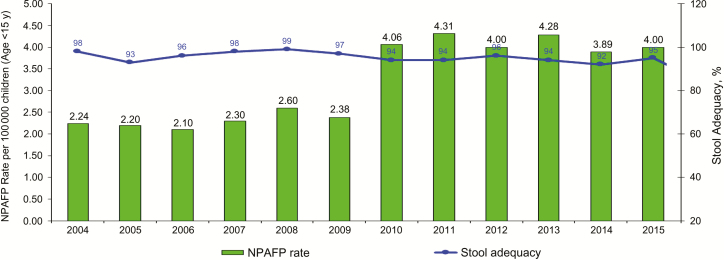
Data collected on polio surveillance indicators by the World Health Organization South Sudan in 2004–2015. Abbreviation: NPAFP, nonpolio acute flaccid paralysis.

In addition to the critical technical support provided by implementing partners and non-governmental organizations, the STOP program has positively contributed to the overall performance of the South Sudan EPI program and its workforce capacity. The 2013 STOP global evaluation team documented that the long-term impact of the STOP program on South Sudan EPI program (specifically on polio eradication and other health programs) is credited to the continuous deployment of STOP volunteers to the same location [7]. Within the first year of STOP deployment, the annualized nonpolio AFP rate, one of the 2 major core indicators of polio surveillance performance, increased from 2.38 to 4.31 per 100000 children <15 years old (Figure 1). That rate remained high since 2010 despite an evacuation of the STOP volunteers for 3 months in 2012 due to security issues in country. The high performance of the South Sudan EPI on polio surveillance and RI indicators is a testament to the active role of the STOP volunteers in strengthening surveillance activities (eg, intensified active case search) and training health workers in AFP surveillance and reporting and other related programmatic areas. Furthermore, the STOP program serves as a platform to build public health capacity among participating countries. STOP volunteers tend to join a public health organization or the MOH after completion of their assignments [7].

The STOP program is a hybrid of capacity-building and capacity-filling strategies for health systems strengthening. Of 17 STOP volunteers interviewed in South Sudan in 2013, 9 reported most of their time spent on capacity-filling activities (eg, updating microplans, active surveillance search), compared to 8 who focused on capacity building (eg, on-the-job training) [7]. Although the STOP program has proved to have a strong and positive impact on the workforce capacity and the overall performance of the South Sudan EPI, its impact is contingent on continuous deployment of STOP volunteers to the same locations. The reliance of the South Sudan EPI on this time-limited program and overall progress toward global polio eradication highlighted an urgent need to build sustainable indigenous capacity within the South Sudan EPI program by developing a strong national workforce at all operational levels. One key recommendation from the 2013 Polio Technical Advisory Group meeting was for South Sudan to develop a long-term plan for transitioning key technical support from external partners to national staff as part of the Polio Eradication and Endgame Strategic Plan, 2013–2018 [8].

## TRANSITIONING FROM THE STOP PROGRAM TO A SUSTAINABLE NATIONAL IMMUNIZATION PROGRAM

### Project Design and Implementation

In collaboration with WHO’s South Sudan country office, the MOH developed a proposal in 2013 outlining the overall goal and objectives of a workforce capacity development project, which estimated ongoing staffing needs and outlined essential EPI-related functions that would need to be strengthened to build a functional national program. The proposal was later presented to the CDC and UNICEF for potential collaboration. This initiative led to a strong multilateral partnership to strengthen workforce capacity for the South Sudan EPI.

**Figure 2.**
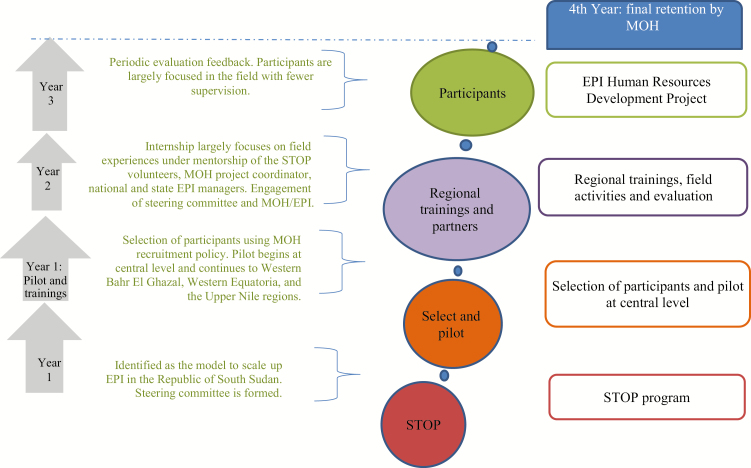
Strategic framework of Expanded Program on Immunizations (EPI) human resources plan for South Sudan. 2015–2018. Abbreviations: MOH, Ministry of Health; STOP, Stop Transmission of Polio.

In collaboration with the CDC, WHO, UNICEF, and the African Field Epidemiology Network (AFENET), the MOH launched the Human Resources Development for South Sudan EPI to achieve polio endgame milestones in 2015. The overall goal of this project is to contribute to the development of an adequately staffed and trained EPI workforce in South Sudan at the central and state/administrative levels. Its aim is to train 56 South Sudanese for the national and state levels on RI, VPD surveillance, SIAs, data management, communication, and cold-chain logistics. During the project, one of the primary roles of the STOP volunteers would be to provide ongoing mentorship to the program’s participants. Figure 2 shows the implementation phases of this 3-year project.

The MOH estimated approximately 8 vacant positions at the national level and 6 vacant positions at each state/administrative area level within the EPI core functions (eg, EPI operations officer, VPD surveillance officer, data manager, communication officer, and SIAs officer). The intention is for those trainees who successfully completed the program to be vested as full-time employees of the EPI unit within the MOH for ≥3 years. It is expected that >85% of the vacant positions at the national level will be filled (approximately 7 of 8 participants). The remaining 48 trainees will be equally distributed among the 12 states/administrative areas; therefore, 50%–67% positions will be filled (3–4 of 6).

### Recruitment

An AFENET technical advisor was recruited to oversee the project and to provide mentorship and supervision to the program’s participants. In collaboration with MOH, WHO, and CDC, the technical advisor is also responsible for the development and implementation of the training curriculum based on desired competencies and for providing technical guidance and direction to the participants, including refresher training as needed.

Participants are recruited from their respective state of residence in coordination with their state MOH levels, WHO, UNICEF, and the in-country technical advisor. Overall, 8 mentees will be recruited from the central level and 3–5 mentees for each state/administrative area depending on agreed-on EPI needs (Table 1). Given the political landscape of various regions/states and the MOH recruitment policy, the recruitment process requires flexibility to adjust and adapt to different settings. As of September 2016, 40 of the 56 total participants have been recruited and have started their assignments. Recruitment is ongoing for the remaining 16. The participants already in place include physicians, clinical officers, epidemiologists, public health officers, and nurses. Although most participants have field experience in management of healthcare interventions in the MOH and/or humanitarian agencies, some have no background in immunization or public health.

**Table 1.** Number of Participants at the Central and State/Administrative Levels (MOH)

State/Administrative Area	Participants, No.					
	RI Operations Officer	SIAs Officer	VPD Surveillance Officer	EPI Data Officer	C4D Officer	Total
Jubek/Yei River/Terekeka (Central Equatoria State)	1	1	1	1	1	5
Imatong/Lomurnyang (Eastern Equatoria State)	1	1	1	1	0	4
Gbudwe/Maridi/Amadi (Western Equatoria State)	0	1	1	1	1	4
Wau/Lol (West Bahr Ghazaal)	1	1	1	1	0	4
Aweil/Aweil East (North Bahr Ghazaal)	1	1	0	1	1	4
Gogrial/Tonj/Twic (Warrap)	1	0	1	1	1	4
Gok/Eastern Lakes/Western Lakes	0	1	1	0	1	3
Abyei	1	1	1	1	0	4
Jonglei/Eastern Bieh/Wetern Bieh	1	1	0	1	1	4
Latjor/Eastern Nile/Western Nile	1	1	0	1	1	4
Ruweng/North Liech/South Liech (Unity)	1	1	0	1	1	4
Boma (formerly Greater Pibor administrative area)	1	1	1	1	0	4
Central MOH	1	1	1	1	1	8^a^
Total	11	12	9	12	9	56^a^

Abbreviations: C4D, communication-for-development; EPI, Expanded Program on Immunization; MOH, Ministry of Health; RI, routine immunization; SIA, supplemental immunization activities; VPD, vaccine-preventable disease.

^a^The 3 additional mentees at the national level are 2 cold-chain officers and 1 EPI logistics officer.

### Training

Training is ongoing by state/administrative level and adjusted for different recruitment timelines. All recruited participants have received a 1-week comprehensive induction training on EPI regardless of their background in immunization and/or public health. Additional training is provided based on their areas of specialty (Table 2). The contents of the 1-week induction training included a general overview of immunization; South Sudan EPI–targeted diseases; the country’s recommended immunization schedule; management of cold-chain equipment; managing vaccines and logistics; introduction to microplanning; planning and implementing SIAs; introduction to communication for development; monitoring, supervision, and evaluation of EPI performance; and VPD surveillance. Through the application of adult learning principles, a mixed training approach uses classroom and field experiences to reinforce specific classroom trainings. The trainings are facilitated by WHO, UNICEF, CDC, and AFENET.

**Table 2.** Training Focus on 7 Core EPI Areas

Core EPI Area	Level
Routine immunization operations	National; state/administrative area
VPD surveillance	National; state/administrative area
SIAs campaigns	National; state/administrative area
EPI data management	National; state/administrative area
EPI communications	National; state/administrative area
Cold-chain maintenance	National
Supply chain logistics	National

Abbreviations: EPI, Expanded Program on Immunization; SIAs, SIA, supplemental immunization activities; VPD, vaccine-preventable disease.

The learning experience of the participants is not homogeneous, but it is dependent on geographic location, areas of specialty, and response to specific events, such as occurrence of a measles outbreak in a given area. The second and third years of this project will focus on reinforcing acquired competencies of the participants. Ad hoc classroom refreshers and competency-based trainings are provided as needed.

### Field Assignments and Mentorship

On completion of the 1-week induction training, the participants are deployed to their assigned sites at the central, state, and administrative levels of the MOH. They receive weekly on-the-job training while in the field, focusing on individual competencies. Field assignments and training topics vary based on the participant’s core EPI focus. For example, a participant whose concentration is RI would receive training on the “reaching every district” approach and EPI service delivery microplanning and would be expected to develop locally relevant microplans and plans for program monitoring and supervision. In contrast, participants whose focus is on cold-chain management would work on the cold-chain maintenance, develop cold-chain replacement and expansion plans, and update cold-chain equipment inventories. Participants are required to provide weekly updates to the technical advisor on their progress with specific assigned tasks.

STOP volunteers and other WHO EPI consultants serve as technical mentors for activities, such as SIAs, RI, case-based surveillance for VPDs, and EPI monitoring. Similarly, UNICEF staff and STOP volunteers serve as technical mentors in EPI communication, cold-chain management, and effective vaccine management. All state-level field mentorship activities are under the supervision of the MOH project coordinator, national and state EPI managers, and the technical advisor. Bill & Melinda Gates Foundation representatives in the states of Jonglei and Upper Nile also provide mentorship to participants.

### Progress to Date

Since the inception of this project, 8 central-level participants were deployed to the field to participate in the meningococcal vaccine SIAs. Furthermore, both central and state-level participants have supported measles outbreak responses in the states of Agok, Mayom, and Gogriel by collecting surveillance and case-based data, creating line lists from selected state and county hospitals to inform response planning, and monitoring postoutbreak response.

Mentoring workshops on preselected immunization-related topics have been conducted weekly. Examples of topics for these weekly mentoring workshops include measles outbreak response and risk analysis, the role of national immunization technical advisory group operations, the switch from trivalent to bivalent oral poliovirus vaccine, sentinel site vaccine utilization monitoring, EPI operations microplanning, and competency-specific training (eg, communication).

The participants have applied competencies developed through these workshops in several ways. For example, the workshop focusing on the switch from trivalent to bivalent oral poliovirus vaccine prepared the participants to support activities related to the switch. Training in microplanning for EPI operations fed into the development of context-relevant microplans to boost EPI coverage required by Gavi, the Vaccine Alliance’s health system strengthening funding. Four communication-for-development participants field-tested a defaulter tracing interview tool aimed at generating behavioral surveillance data to be used for an upcoming project on communication. The communication-for-development, SIA, and RI operations participants participated in a UNICEF-specific training on development of a communication strategy for conflict-affected areas. Cold-chain technician participants have received certified training in installation and maintenance of solar direct-drive refrigerators. As a result, they supported installation of 10 solar refrigerators in the Greater Central Equatoria and completed assessment of cold-chain equipment in that area. They have already repaired 22 EPI refrigerators, 4 ice-pack freezers, and 3 deep freezers, and will continue to repair all EPI refrigerators and freezers in a systematic and scheduled maintenance plan being developed by the MOH.

Implementation of this project and its progress are being monitored routinely and will culminate with a yearly program assessment to evaluate implementation and progress with planned activities. The monitoring and evaluation plan and framework as well as standardized tools are being finalized to capture an in-depth understanding of participants’ progress with skills and knowledge acquisition in their core competency areas. Results of monitoring and evaluation activities will inform project implementation, including activities of the participants in the field.

## CHALLENGES

Implementing this project has not been an easy feat. Ongoing security issues have hampered movement to different regions of the country and even within states. Consequently, some implementing partners have not been able to visit the country since implementation to provide direct technical support. Moreover, the recent creation of 16 additional states has substantially changed the recruitment process and scope of the project. This change has led to a delay in the recruitment timeline. Constraints imposed by ethnic and tribal tensions have also restricted movement of participants. For example, participants from Central Equatoria state cannot safely move to Terekeka state, which was recently created to be a state dominated by the Mundari ethnic group.

The Republic of South Sudan has been identified by the GPEI as high risk for poliovirus importation from neighboring countries. In the event of an importation, field activities will be modified to reflect the current situation on the ground. The transition plan from a polio-free world to other health programs is not yet finalized in South Sudan; however, this project will fit well into the transition plan.

## WAY FORWARD

The principle objective of this project is to develop a qualified, skilled, and sustainable cadre of immunization public health workers. The project is designed to build on the legacy of the international STOP program to develop and sustain national workforce capacity. Its approach is collaborative and leverages a broad range of partnerships and expertise to advance the South Sudan EPI’s main objectives and priorities (including polio eradication goals and objectives), respond to other public health emergencies, and strengthen RI.

The project highlights the feasibility of building national workforce capacity in a country with multiple challenges, including security issues and ethnic conflicts. It serves as a platform to share and exchange knowledge, ideas, and experience on strengthening human resources capacity, using nationals as a sustainable approach instead of international consultants to temporarily fill human resource gaps. Given that this project is still in its infancy stage, lessons learned from it will be documented at a later date.

Depending on the evolution of this project and the political stability of the country, there may be a possibility to expand the project to build future capacity at the county/district levels, where most operations and actions for improving immunization coverage take place. This would also enable provision of enhanced RI support to hard-to-reach and high-risk areas. The long-term sustainability of the project is clearly dependent on the country’s political stability and the degree of political and financial commitment to the immunization program by the government of South Sudan. Nevertheless, stakeholders and the team remain committed to the success of this project and to the overall goal of building a strong immunization public health workforce capacity in South Sudan.

## Notes

***Supplement sponsorship.*** This work is part of a supplement coordinated by the Task Force for Global Health with funding provided by The Bill and Melinda Gates Foundation and the Centers for Disease Control and Prevention.

***Potential conflicts of interests***. All authors: No reported conflicts.

## References

[1] Ministry of Health, Government of Southern Sudan Health policy for the Government of Southern Sudan, 2006–2011 http://www.africanchildforum.org/clr/policy%20per%20country/south%20sudan/ssudan_health_2006-2011_en.pdf Accessed 18 July 2016.

[2] Ministry of Health, Government of Southern Sudan Basic package of health and nutrition services for Southern Sudan, 2009 http://www.unicef.org/southsudan/South_Sudan_Basic_package_of_health_services.pdf Accessed 18 July 2016.

[3] Ministry of Health, Republic of South Sudan Comprehensive multi-year plan for immunization services in Southern Sudan, 2007–2011. (Unpublished).

[4] Ministry of Health, Republic of South Sudan External EPI program review in South Sudan, 2011. (Unpublished).

[5] World Health Organization. Surveillance data collected by WHO South Sudan, 2004–2015. Unpublished.

[6] Centers for Disease Control and Prevention (CDC). The Global Polio Eradication Initiative Stop Transmission of Polio (STOP) Program—1999–2013. MMWR Morb Mortal Wkly Rep 2013; 62:501–503. 23784015 PMC4604894

[7] Stop Transmission of Polio (STOP) Global Evaluation, 2013 Stop Transmission of Polio Program. (Unpublished).

[8] Global Polio Eradication Initiative. Polio eradication and endgame: strategic plan 2013–2018 http://www.polioeradication.org/Portals/0/Document/Resources/StrategyWork/PEESP_EN_US.pdf Accessed 7 September 2016.

